# The Surgeon-General of the Federal Army

**Published:** 1865-04

**Authors:** 


### THE SURGEON-GENERAL OF THE FEDERAL ARMY.

By Editor of London Lancet.

We gave lately some details of the charges brought against the Surgeon-General of the Federal Army, and the finding of the Court Martial which dismissed him from that high position on the grounds of corrupt practice. The charges against him were of three kinds: they alleged acts in excess of authority; they charged personal corruption and intent to aid others to defraud the Government, and wilful falsehood. There were many circumstances connected with the Court-Martial by which the sentence was pronounced which indicated that its finding could not be accepted as satisfactory without further inquiry. In the first place, it appeared that the Surgeon-General had, prior to the assembling of the Court, been ordered to a distant part of the continent, and there charged with personal labors evidently designed to remove him from the scene of inquiry, and to inflict upon him a personal indignity, while they prevented him from preparing a defence. The Surgeon-General has now issued a statement of the causes that led to his dismissal, with a review of the evidence adduced before the Court, which is perhaps one of the most remarkable documents ever published by a public officer, and discloses a state of things which could hardly be supposed to exist, even amid such a combination of despotism and mobocracy as the civil war has produced in Northern America.

Dr. Hammond was appointed at the pressing instance of the American Sanitary Commission and of General McClellan and others. His appointment was unpopular with the Cabinet, but it could not be resisted. Two days after his appointment he was sent for by Mr. Stanton, Secretary of War, and at the close of a brief conversation was ordered by that gentleman "to leave his office immediately," from which time he of course "never entered it except upon strictly official business." This was a startling commencement of official relations. Their subsequent communications were of a corresponding character; incessant reprimands; worrings; orders to report upon "what authority" this thing or the other thing was done. Presently the Secretary ordered a Commission to inquire into the conduct and affairs of the Department, naming for the purpose as President a person known to be a particular enemy of Dr. Hammond, with whom he had publicly quarreled, and who had sworn "to be revenged upon him." Dr. Hammond says: "The examination by this Commission was entirely *ex parte*. I was never called on for an explanation of any kind, and no witness was allowed to say anything which could be interpreted as favorable to me. I was never present at any session of the Commission. On the contrary, as soon as they had fairly entered upon their work, I was ordered to locate myself in the Department of the Gulf till further orders. I was therefore relieved from the charge of the Medical Bureau in Washington, and on the 30th of August, 1863, left that city. I have never been in charge of the Department since."

This Commission made a report, for a copy of which Dr. Hammond of course applied; but no notice was taken of the application, and he has never seen the report to this day. Meantime he was exiled to a distant department, and deprived of his authority as Surgeon-General. He made urgent application for a court-martial. "On the 15th of January," he states, "I arrived in Washington, in accordance with permission, granted only after I had received a severe fall, by which I was paralysed for several months; and on the 17th I was placed in arrest, and ordered to be tried by a court-martial, which was to meet on the 19th. I received the announcement with joy. I was confident that no unprejudiced court would convict me of wrong-doing in the face of the evidence of my innocence which would be presented," However, he was found guilty of corruption and of excess of authority. Reading the evidence which Surgeon-General Hammond now produces, it is impossible not to concur with him in affirming that the finding is highly dishonorable to the Court which convicted, and in no respect to himself. It is absolutely disproved by the documents which he produces; and we have no hesitation in declaring that Dr. Hammond stands now acquitted in the face of Europe and before his profession, and that his judges are condemned.

There are other circumstances disclosed by his statement which are of the most astounding character, and reflect deeply upon the conduct of the inquiry and the state of morality in Government departments under the present *regime* in Washington. In the course of the inquiry, a package of letters which had been stolen from his office was returned to his counsel, under cover, with the following letter:—

"Circumstances have placed the enclosed papers in my control, and I know where there are others which bear strongly in General Hammond's favor, and which have been secretly taken from his office. I will obtain them if possible. He has been and now is conspired against. I cannot remain silent while a great wrong is attempted. I dare not tell you *how* I got those papers. *I* did not steal them. I know you will do what is right with them. My only object is JUSTICE."

"There were events," writes Dr. Hammond, "connected with the return of those letters to which I do not more specifically refer now, as I hope to be able ere long to connect the several links into a complete chain of evidence. I will only say that no doubt exists that these letters had in part been stolen from my office, and been mixed by some one with letters which had been sent by me to Dr. Cooper and to the War Department. In all, the package contained forty-nine papers. They were of such a character as showed that my office had been ransacked from top to bottom, and even the private drawers of my desk invaded. It was doubtless in one of these raids that Dr. Smith's money was taken."

When the history of this war is written, this defence of Dr. Hammond will be amongst the documents worthy of the attention of the historian. Meantime we congratulate the Surgeon-General on having cleared his character, and supported his integrity by the strongest documentary evidence.

